# Improved Genetic Profiling of Anthropometric Traits Using a Big Data Approach

**DOI:** 10.1371/journal.pone.0166755

**Published:** 2016-12-15

**Authors:** Oriol Canela-Xandri, Konrad Rawlik, John A. Woolliams, Albert Tenesa

**Affiliations:** 1 The Roslin Institute, Royal (Dick) School of Veterinary Studies, The University of Edinburgh, Easter Bush Campus, Midlothian, Scotland, United Kingdom; 2 MRC HGU at the MRC IGMM, University of Edinburgh, Western General Hospital, Edinburgh, United Kingdom; Stanford University School of Medicine, UNITED STATES

## Abstract

Genome-wide association studies (GWAS) promised to translate their findings into clinically beneficial improvements of patient management by tailoring disease management to the individual through the prediction of disease risk. However, the ability to translate genetic findings from GWAS into predictive tools that are of clinical utility and which may inform clinical practice has, so far, been encouraging but limited. Here we propose to use a more powerful statistical approach, the use of which has traditionally been limited due to computational requirements and lack of sufficiently large individual level genotyped cohorts, but which improve the prediction of multiple medically relevant phenotypes using the same panel of SNPs. As a proof of principle, we used a shared panel of 319,038 common SNPs with MAF > 0.05 to train the prediction models in 114,264 unrelated White-British individuals for height and four obesity related traits (body mass index, basal metabolic rate, body fat percentage, and waist-to-hip ratio). We obtained prediction accuracies that ranged between 46% and 75% of the maximum achievable given the captured heritable component. For height, this represents an improvement in prediction accuracy of up to 68% (184% more phenotypic variance explained) over SNPs reported to be robustly associated with height in a previous GWAS meta-analysis of similar size. Across-population predictions in White non-British individuals were similar to those in White-British whilst those in Asian and Black individuals were informative but less accurate. We estimate that the genotyping of circa 500,000 unrelated individuals will yield predictions between 66% and 82% of the SNP-heritability captured by common variants in our array. Prediction accuracies did not improve when including rarer SNPs or when fitting multiple traits jointly in multivariate models.

## Introduction

Phenotypic prediction of complex traits from genomic data could transform clinical practice by enabling tailored treatment and targeted disease screening programs based on the genetic make-up of the individual, and by facilitating more efficient allocation of resources within the health systems [1–3]. Ultimately, it would help to understand the underlying disease mechanisms and open the targeted search of specific solutions based on this knowledge. With this in mind, large efforts and investments in the past years have been directed towards generating genotypic and phenotypic data for identifying individual genetic variants associated with different traits through genome-wide association studies (GWAS) [4]. Although using this approach a large number of susceptibility variants for many diseases have been identified, the strategy has several limitations. First, the accuracy of prediction has been disappointingly low for traits affected by a large numbers of susceptibility variants [3]. Second, the approach of identifying one single nucleotide polymorphism (SNP) at a time and including such newly identified SNPs in the prediction models as and when they are identified is unpractical if one wishes to use genetic tests for multiple traits because the composition of each trait’s genetic test would need to be continuously updated and each trait would require its own SNP panel. Third, statistical considerations and simulation studies have shown that the accuracy of prediction for complex traits increases by modelling all available SNPs simultaneously [5].

Recent studies have shown that SNP arrays containing common genetic variants capture a substantial amount of the genetic variation for each trait and that the contributing SNPs have effects generally too small to be detected with current GWAS sample sizes due to the stringent genome-wide significance levels applied [6–8]. Furthermore, we have previously shown through simulations that the size of the studies that have estimated heritability from SNP arrays have been too small to properly estimate SNP effects for accurate phenotypic prediction [9]. However, the availability of large genotyped cohorts for which individual-level data is available, e.g. the UK Biobank [10,11], combined with new and powerful computational tools [9] capable of fitting complex statistical models to big datasets and access to high-performance computational infrastructure has the potential to provide accurate SNP effects for genomic prediction.

We show that modelling individual-level data of circa 110,000 individuals can lead to accurate predictions across multiple traits by jointly fitting the SNPs of a single array of common SNPs. We first focused on human height, a highly heritable quantitative trait commonly used as a model in the study of the genetic architecture of complex traits [6,7,12] and one of the traits for which most contributing loci have been identified to date. To increase the generality of our findings, we then selected four obesity related traits—BMI, body fat percentage, waist-to-hip ratio (WHR) and basal metabolic rate (BMR). The obtained predictions significantly improve on the accuracies of models derived from summary statistics obtained from large GWAS meta-analyses and in turn may ease clinical implementation and direct-to-consumer genetic testing, as well as improve the accuracy of the predictions as the sample sizes of the training datasets increase.

## Results

For our analyses, we used the 152,736 genotyped individuals available from the UK Biobank cohort [10]. After applying stringent quality control criteria, we divided our sample into White-British (123,847 individuals) and non White-British (27,685 individuals), the latter including individuals from different ethnic backgrounds (Online Methods and S1 Fig). We divided the White-British further into a group of 114,264 unrelated individuals with a relatedness below 0.0625 (i.e. less related to each other than second cousins once removed), another group of 9,583 individuals that had at least one relationship above 0.0625 with the unrelated White-British group, and a group of self-reported White-British (Online Methods and S1 Fig). We modelled 319,038 common SNPs, that is, variants with a minor allele frequency (MAF) >0.05 that passed our genotype quality control.

In order to jointly estimate the additive effects of all SNPs we fitted them as random effects in a Mixed Linear Model (MLM) on the training population, with gender and age as fixed effects (Online Methods). As the computational requirements of MLM fitting rapidly increases with incrementing sample sizes, we used DISSECT [9] (https://www.dissect.ed.ac.uk), a software specifically designed to perform genomic analysis in large supercomputers. Each analysis required ~1h of computing time on the ARCHER supercomputer, harnessing the joint power of 1,152 processors. Using the jointly estimated SNP effects (SNP-BLUPs) to predict the genetic value of individuals in an independent validation dataset (Online Methods), we computed the prediction accuracy as the correlation, r, between these predicted genetic values and the phenotypes corrected for gender and age.

We used the 114,264 unrelated White-British individuals to train the prediction models and assessed the validity of the within-population predictions using the 9,583 related White-British individuals and the 12,640 self-reported White-British individuals. In order to avoid potential overfitting, and the associated inflation of accuracies when performing predictions [13], the validation datasets were not used in any way during the training of the models. Prediction accuracy in the self-ported White-British ranged from 0.51 (95% CI 0.49–0.52) for height to 0.20 (95% CI 0.19–0.22) for WHR (Table 1). We evaluated whether prediction accuracies can be further improved by using more complex models where SNPs are grouped in two groups as a function of their effect size (see Phenotype prediction using a two variance components model section of the Online Methods). The accuracy for height improved to 0.55 (95% CI 0.53–0.56) despite a small reduction in the estimate of heritability. However, accuracies for the other traits decreased. The accuracies we obtained represent between 75% and 46% of the maximum achievable given the estimated SNP-based heritabilities of the traits in unrelated White-British, i.e., 0.53 (SE = 0.004), 0.26 (SE = 0.005), 0.26 (SE = 0.005), 0.20 (SE = 0.005) and 0.31 (SE = 0.005) for height, body fat percentage, BMI, WHR, and BMR, respectively (Online Methods). As expected, phenotypic prediction for relatives was more accurate than that for the self reported White-British, likely because their phenotypes and genotypes are more correlated to the training samples. The robustness of our within-population predictions were confirmed using 10-fold cross-validation [14] within the unrelated White-British participants (S1 Table).

**Table 1:** Prediction accuracies on related White-British and self-reported White-British.

Traits	self-reported White-British (95% CI)	related White-British (95% CI)
**Height**	0.51 (0.49–0.52)	0.53 (0.52–0.55)
**Body fat percentage**	0.27 (0.25–0.29)	0.28 (0.26–0.30)
**BMI**	0.25 (0.24–0.27)	0.27 (0.26–0.29)
**WHR**	0.20 (0.19–0.22)	0.23 (0.21–0.25)
**BMR**	0.32 (0.31–0.34)	0.34 (0.32–0.36)

We also investigated to what extend across-population prediction was feasible. To this end, we further subdivided the non White-British subset by self-reported ethnic background. Excluding ethnicities with less than 1,000 individuals and removing outliers resulted in 7,541 White individuals who did not self-report as White-British, 1,954 Asian or Asian-British individuals, and 1,591 Black or Black-British individuals (Online Methods). Predictions obtained in the White cohort (Table 2) were almost as accurate as to those obtained in the self-reported White-British cohort. This could have been potentially explained by the inclusion of Irish individuals inside the White non British cohort. However, when the White non British group is subdivided in Irish and White non-British non-Irish groups, the accuracies dropped slightly (S2 Table) but were consistent with those obtained in Table 2. Predictions for the other two ethnicities remained considerable but lower than within-population predictions, especially for Black or Black British as expected from the genetic distance between populations (S2 Fig), indicating that predictions may benefit from within-ethnic group tailored models.

**Table 2:** Across-population prediction accuracies.

Traits	White non British (95% CI)	Asian/Asian-British (95% CI)	Black/Black-British (95% CI)
**Height**	0.50 (0.48–0.51)	0.34 (0.30–0.38)	0.18 (0.14–0.23)
**Body fat percentage**	0.26 (0.24–0.28)	0.21 (0.16–0.25)	0.12 (0.07–0.17)
**BMI**	0.25 (0.23–0.27)	0.22 (0.18–0.26)	0.11 (0.06–0.16)
**WHR**	0.21 (0.19–0.23)	0.14 (0.10–0.19)	0.07 (0.02–0.12)
**BMR**	0.32 (0.30–0.34)	0.22 (0.18–0.26)	0.12 (0.07–0.17)

**Fig 1:**
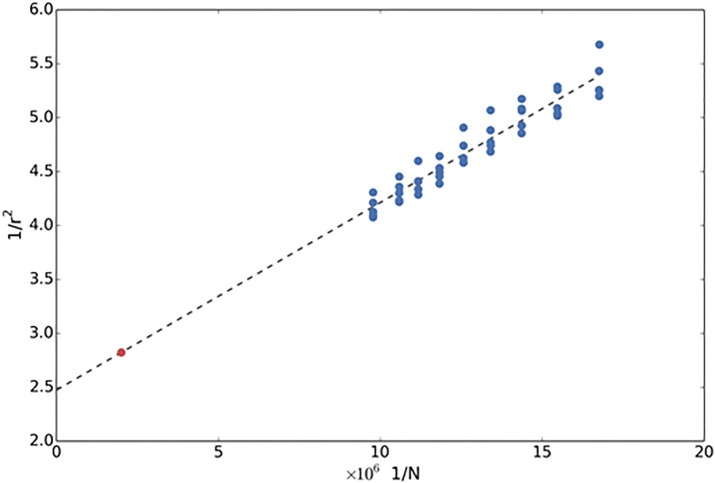
Prediction accuracy as a function of sample size for height. Inverse of the square of the prediction accuracy as a function of the inverse of the training sample size. Blue dots indicate prediction accuracies achieved on several trials. The dashed straight line shows the linear regression fit to the blue dots. The regression intercept indicates the maximum accuracy achievable using common variants represented in the array. The red dot is the expected prediction accuracy with a training sample size of 500,000 individuals.

Although samples sizes for training the models will increase in the future, it is unlikely that they will increase indefinitely. Therefore, we argued that it would be useful to know what sample size would be required to exploit all the genetic variation captured by the SNP array. To gauge this, we computed prediction accuracies for samples of decreasing size, by randomly subsampling the unrelated White-British individuals (Online Methods). Our data fitted very well to a well-known theoretical model [15]. Our results suggest that prediction accuracies for height will reach 0.6 (SE = 0.02) when training the models using ~500,000 individuals (Fig 1), the sample size planned to be genotyped UK Biobank in the near future. This prediction accuracy would represent 82% of the maximum accuracy possible given the explained heritability. Similarly, we estimate that genetic prediction models for BMI, WHR, body fat percentage and BMR will reach prediction accuracies of 0.36 (SE = 0.05), 0.29 (SE = 0.03), 0.37 (SE = 0.03), and 0.42 (SE = 0.03), respectively (S3 Fig).

**Fig 2:**
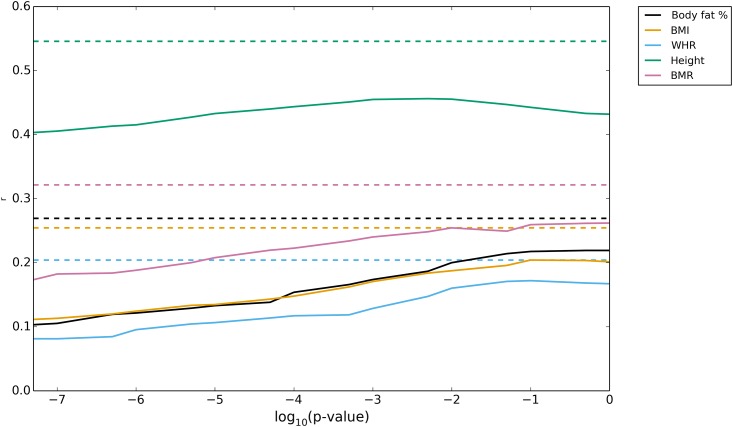
Prediction accuracies from GWAS analyses. Predictions on self reported White-British obtained using independently estimated SNP effects from a GWAS. We plot the accuracies obtained for subsets of SNPs selected based on a particular p-value threshold against this threshold value. Different colours indicate different traits. Dashed lines indicate maximum accuracies obtained when the effects of all SNP were estimated jointly (SNP-BLUP) using DISSECT.

We compared the SNP-BLUP prediction accuracies on self-reported White-British with predictions obtained using a GWAS approach on the same dataset. In a GWAS, each SNP is fitted independently and we computed the prediction accuracies by selecting a list of independent SNPs at different levels of statistical significance [6,12] ranging from 5*10^−8^ to 1 (i.e. all independent SNPs included) (Online Methods). The maximum accuracies obtained by the SNP-BLUP approach are up to 25% better (i.e. explain up to 55% more variance) and are consistently larger than the maximum obtained using the selected SNPs from the GWAS approach with a p-value over the optimal threshold (Fig 2). This is the case, despite the potential of overfitting and hence inflation of prediction accuracies in the GWAS approach, due to the use of the validation population for selecting the optimal p-value threshold. Interestingly, while for height, the maximum for the accuracy is achieved inside the range of tested p-value thresholds; this is not the case for the other traits, where the best predictions are obtained at the boundary where all independent SNPs are included. This may suggest that the genetic architecture for these traits differs from that of height.

Finally, we studied whether we can improve the accuracies by both, including rarer variants, or by fitting more than one trait together in a multivariate model. Although inclusion of rare variants increases the heritability estimates slightly (S3 Table), the prediction accuracies remain nearly identical to those obtained using only common variants (Table 1 and S4 Table). This could be due to the difficulty of estimating their effects, even with the large sample sizes used here. Finally, we performed multivariate analyses on the traits divided into two groups based on their pairwise genetic correlation (see S4 Fig). That is, we fitted both, a bivariate model with height and BMR and a trivariate model with BMI, WHR, and Body fat percentage (Online Methods). In both cases, prediction accuracies did not show a significant improvement compared to those obtained from fitting each trait separately (Table 1 and S5 and S6 Tables).

## Discussion

Our results confirm previous findings that many variants with small effect can explain a large proportion of the genetic variance. Due to several factors, this part of the genetic variance has so far remained largely unexploited for phenotypic prediction. These factors include the statistical methods used, the available sample sizes, and computational software available to analyse the data. However, as we have shown, predictions which are significantly more accurate can be obtained by increasing sample sizes and using powerful computational approaches to jointly estimate all SNP effects. The accuracy of the SNP-BLUP predictor for height is 68% larger (i.e. a 184% relative improvement in variance) than that obtained by GWAS meta-analysis hits based predictors developed through large collaborations, which used similar number of training samples [12]. If instead of using GWAS hits for prediction, the SNPs are selected based on an optimal p-value threshold obtained using the validation population, the accuracy of the SNP-BLUP based predictors improves by 50% (i.e. 124% more variance explained) when compared with a previous work that used a sample size similar to ours [12], and it is 32% (i.e. 75% more variance explained) larger when compared with previous work that used a training sample size ~250% larger than ours [6]. For BMI, which has a lower SNP heritability, the SNP-BLUP predictor explains slightly more variance than previous GWAS based predictors, despite our training sample being almost ~3 fold smaller [16]. However, taking into account our own GWAS based prediction results, the improvements we report in prediction accuracy (compared to previous GWAS based results [6,12,16]) could potentially be affected by several factors in addition to the method used. For instance, it is possible, that the genetic distance between the training and validation populations in our data is smaller than in previous studies. Another difference between our study and previous ones is that we used individual level genotype data, that is, the accuracy of prediction may be reduced when performing meta-analyses by combining data from heterogeneous studies (e.g. that may use different arrays or study dependent covariate variables). The possibility of our training and testing population being more similar is somehow mitigated because we are using a set of genotypic outliers (the self-reported White-British) from our core population as one of our validation sets. Prediction in this subset may be considered a good indicator for the accuracy that one may expected to achieve in the general White-British population. For height, our SNP-BLUP prediction accuracies are close to the maximum achievable given the estimates of the explained heritable component, and we predict that they will approach this maximum even more when the number of samples increases (e.g. when the UK Biobank is fully genotyped). Furthermore, the accuracy observed for White non-British individuals was very close to the accuracy for White-British individuals, further suggesting that accuracy in the general White-British population would be similar. We have also shown that, at least for the traits and methods studied here, accuracies do not significantly improve when rarer variants genotyped in our array are included in the models. This was the case despite slight increases in estimates of heritability. Finally, we demonstrated that more complex models have the potential to further improve prediction accuracies, although our results also indicate that the optimal model may be trait specific. In conclusion, the presented results support our initial hypothesis and suggest a promising future for genomic prediction of complex traits.

## Methods

### Genotype quality control

For our analysis, we used the data for the genotyped individuals in phase 1 of the UK Biobank genotyping program. 49,979 individuals were genotyped by using the Affymetrix UK BiLEVE Axiom array and 102,750 individuals by using the Affymetrix UK Biobank Axiom array. Details regarding genotyping procedure and genotype calling protocols are provided elsewhere (http://biobank.ctsu.ox.ac.uk/crystal/refer.cgi?id=155580). From the overlapping markers, we excluded those which were multi-allelic, their overall missingness rate exceeded 2% or they exhibited a strong platform specific missingness bias (Fisher’s exact test, P < 10^−100^). We also excluded individuals if they exhibited excess heterozygosity, as identified by UK Biobank internal QC procedures (http://biobank.ctsu.ox.ac.uk/crystal/refer.cgi?id=155580), if their missingness rate exceeded 5% or if their self-reported sex did not match genetic sex estimated from X chromosome inbreeding coefficients. These criteria resulted in a reduced dataset of 151,532 individuals. Finally, we only kept the common variants (i.e. with a MAF > 0.05) and those that did not exhibit departure from Hardy-Weinberg equilibrium (P < 10^−50^) in the unrelated (subset of individuals with a relatedness below 0.0625) White-British cohort (see below).

### Ethnicity

The UK Biobank samples are from individuals of diverse ethnicities. To define the White-British cohort, we performed a Principal Components Analysis (PCA) of all individuals passing genotypic QC using a linkage disequilibrium (LD) pruned set of 99,101 autosomal markers (http://biobank.ctsu.ox.ac.uk/crystal/refer.cgi?id=149744) that passed our SNP QC protocol. The related and unrelated White-British individuals were defined as those for whom the projections onto the leading twenty genomic principal components fell within three standard deviations of the mean and who identified themselves as White-British. We defined the other removed White-British as self-reported White-British. The other ethnicities were defined using the self-identified ethnic background (http://biobank.ctsu.ox.ac.uk/crystal/field.cgi?id=21000). As we did with White-British individuals, we only retained those individuals whose projections onto the leading twenty genomic principal components fell within three standard deviations of the ethnicity group mean (S2 Fig).

### Phenotype quality control

We defined outliers as males and females that were outside ±3 standard deviations from their gender mean of all the individuals in the UK Biobank, and removed them from the analyses.

### Software

The genotype quality control and data filtering was performed using plink [17] (https://www.cog-genomics.org/plink2). The PCA, MLMs fittings for estimating SNP effects and phenotype predictions were performed using DISSECT (https://www.dissect.ed.ac.uk) on the UK National Supercomputer (ARCHER). DISSECT software is designed to perform genomic analyses on very large sample sizes without the need to perform mathematical approximations by using the power of large supercomputers.

### Software URLs

DISSECT and documentation available at: https://www.dissect.ed.ac.uk

PLINK2 and documentation available at: https://www.cog-genomics.org/plink2

BOLT-LMM and documentation available at: https://data.broadinstitute.org/alkesgroup/BOLT-LMM/

### Phenotype prediction

The effect of all SNPs were estimated together as a random effect using the model,

$$y_{i}=\mu +\sum_{l=1}^{L}x_{il}\beta_{l}+\sum_{j=1}^{M}z_{ij}a_{j}+e_{i},$$

where *μ* is the mean term and *e*_*i*_ the residual for individual *i*. *L* is the number of fixed effects, *x*_*il*_ being the value for the fixed effect *l* at individual *i* and *β*_*l*_ the estimated effect of the fixed effect *l*. *M* is the number of markers and *z*_*ij*_ is the standardised genotype of individual *i* at marker *j*. The vector of random SNP effects **a** is distributed as $\text{N}\left(0,I\sigma_{u}^{2}\right)$. The vector of environmental effects **e** is distributed as $\text{N}\left(0,I\sigma_{e}^{2}\right)$. Defining $\sigma_{g}^{2}=M\sigma_{u}^{2}$, the heritabilities were estimated as $\sigma_{g}^{2}/\left(\sigma_{e}^{2}+\sigma_{g}^{2}\right)$. The inversion of the covariance matrix is where almost all computational resources are used when fitting these models.

The prediction of the phenotype $\hat{y_{i}}$ for the individual *i* was computed as a sum of the product of the SNP effects and the number of reference alleles of the corresponding SNPs:

$$\hat{y_{i}}=\sum_{j=1}^{M}\frac{\left(s_{ij}-\mu_{j}^{*}\right)}{\sigma_{j}^{*}}a_{j},$$

where *s*_*ij*_ is the number of copies of the reference allele at SNP *j* of individual *i*, *M* is the number of SNPs used for the prediction, and *a*_*j*_ the effect of SNP *j*. $\mu_{j}^{*}$ and $\sigma_{j}^{*}$ are the mean and the standard deviation of the reference allele in the training population.

Prediction accuracies were computed as the correlation between the predicted phenotype and the real one after correcting by the estimated effect of the used covariates (e.g. sex and age).

### Phenotype prediction using a two variance components model

The MLM of the previous section assumes that all SNP effects follow a Gaussian distribution with one variance. However this is may not be true. To improve the model we first fitted all SNPs independently using a standard GWAS model,

$$y_{i}=\mu +\sum_{l=1}^{L}x_{il}\beta_{l}+z_{ij}a_{j}^{*}+e_{i}.$$

Here, the parameters are the same as in the previous MLM, and the SNP effect size $a_{i}^{*}$ is estimated independently for each SNP as a fixed effect. We then divided the SNPs into two groups based on their effect size. Specifically, one group of SNPs in the main distribution and a group of outliers, which were defined as SNPs with effect sizes more than 3 standard deviations away from the mean effect across all SNPs. Using these groups, we fit an extended MLM where we assume the SNP effects were distributed in two different Gaussian distributions with a different variance each one,

$$y_{i}=\mu +\sum_{l=1}^{L}x_{il}\beta_{l}+\sum_{j=1}^{M}z_{ij}^{m}a_{j}^{m}+\sum_{k=1}^{K}z_{ik}^{t}a_{k}^{t}+e_{i},$$

where all parameters are the same as in the simpler MLM, but now M and K are the number of SNPs in the main distribution and the two tails, respectively, while $z_{ij}^{m}$ and $z_{ik}^{t}$ are the corresponding genotypes. We fit independent variances for the two groups of SNPs, so that the vector of SNP effects in the main distribution, ${\text{a}}_{j}^{m}$**,** is distributed as $\text{N}\left(0,I{\left(\sigma_{u}^{m}\right)}^{2}\right)$ and the vector of SNP effects in the tails, ${\text{a}}_{k}^{t}$**,** is distributed as $\text{N}\left(0,I{\left(\sigma_{u}^{t}\right)}^{2}\right)$.

### Phenotype prediction using rare variants

We performed predictions using a model similar to that introduced in the previous subsection, but now grouping SNPs based on their MAF. We created three groups: SNPs with MAF > 0.05, SNPs with MAF<0.05 and MAF>0.01, and SNPs with MAF<0.01 and MAF>0.001. Then, we fitted the model:

$$y_{i}=\mu +\sum_{l=1}^{L}x_{il}\beta_{l}+\sum_{k=1}^{3}\sum_{j=1}^{M_{maf_{k}}}z_{ij}^{maf_{k}}a_{j}^{maf_{k}}+e_{i},$$

where now, $M_{maf_{k}}$ is the number of SNPs on the MAF group *maf*_*k*_ and the distribution of the vector of SNP effects ${\text{a}}_{j}^{maf_{k}}$**,** is $\text{N}\left(0,I{\left(\sigma_{u}^{maf_{k}}\right)}^{2}\right)$.

### Multivariate models

We estimated the SNP effects by fitting different traits together in a multivariate model. These models can be expressed as:

$$y_{i}^{t}=\mu^{t}+\sum_{l=1}^{L}x_{il}^{t}\beta_{l}^{t}+\sum_{j=1}^{M}z_{ij}^{t}a_{j}^{t}+e_{i},$$

where the parameters are as in the previous subsections, but now we include phenotypes for different traits, *t*. In this model, the vector of SNP effects for the trait *t*, ***a***^***t***^, has the covariance structure,

$$\left(\begin{array}{c} a^{1}\\ a^{2}\\ \vdots \\ a^{T} \end{array}\right)\sim N\left(0,\left(\begin{array}{cccc} I{\left(\sigma_{u}^{1}\right)}^{2} & I\sigma_{u}^{12} & \cdots & I\sigma_{u}^{1T}\\ I\sigma_{u}^{12} & I{\left(\sigma_{u}^{2}\right)}^{2} & \cdots & I\sigma_{u}^{2T}\\ \vdots & \vdots & \ddots & \vdots \\ I\sigma_{u}^{1T} & I\sigma_{u}^{2T} & \cdots & I{\left(\sigma_{u}^{T}\right)}^{2} \end{array}\right)\right),$$

where ${\left(\sigma_{u}^{t}\right)}^{2}$ is the SNP effects variance for the trait *t*, and $\sigma_{u}^{t_{1}t_{2}}$ is the covariance between traits *t*_1_ and *t*_2_.

### GWAS prediction analysis

GWAS for performing the predictions detailed in Fig 2, were conducted using BOLT-LMM software [18]. For each analysis, the predictions were obtained by using a list of independent associated SNPs at various p-value thresholds. These independent SNPs had been seleted using the PLINK clumping procedure, with an LD-based threshold of r2 > 0.05, and a physical distance threshold of 1 Mb.

### Random subsampling

We computed accuracies for samples of decreasing size, by randomly subsampling 5 of the 10-fold cross-validation subsets used in the within unrelated White-British population predictions (S1 Table).

## Supporting Information

S1 Fig UK Biobank individual splitting. (PNG)

S2 Fig Two first principal components for three different ethnicities. The individuals from different ethnic backgrounds are plotted using different colors.
(PNG)

S3 Fig Prediction accuracy as a function of sample size for four traits. Inverse of the square of the prediction accuracy as a function of the inverse of the training sample size for (**A**) Body fat percentage, (**B**) BMI, (**C**) WHR, and (**D**) BMR. Blue dots indicate prediction accuracies achieved on several trials. The dashed straight line shows the linear regression fit to the blue dots. The regression intercept indicates the maximum accuracy achievable using common variants. The red dot is the expected prediction accuracy with a training sample size of 500,000 individuals.
(PNG)

S4 Fig Genetic and environmental correlations between traits. Genetic and environmental correlations displayed over and below the diagonal, respectively.
(PNG)

S1 Table Prediction accuracies from 10-fold cross validation analysis on unrelated White-British. (DOCX)

S2 Table Prediction accuracies obtained from splitting the White non-British between those that are self-reported as Irish and the remaining. (DOCX)

S3 Table Heritability contribution for each MAF-based group of SNPs. (DOCX)

S4 Table Prediction accuracies obtained when including the computed SNP effects with rarer variants. (DOCX)

S5 Table Prediction accuracies by using SNP effects obtained from a bivariate analysis of height and BMR. (DOCX)

S6 Table Prediction accuracies by using SNP effects obtained from a trivariate analysis of Body fat percentage, BMI and WHR. (DOCX)
