# Supplementary figures and images for: Improved Genetic Profiling of Anthropometric Traits Using a Big Data Approach
Source: PLoS One. 2016 Dec 15;11(12):e0166755. doi: 10.1371/journal.pone.0166755 (PMC5157980; doi:10.1371/journal.pone.0166755)

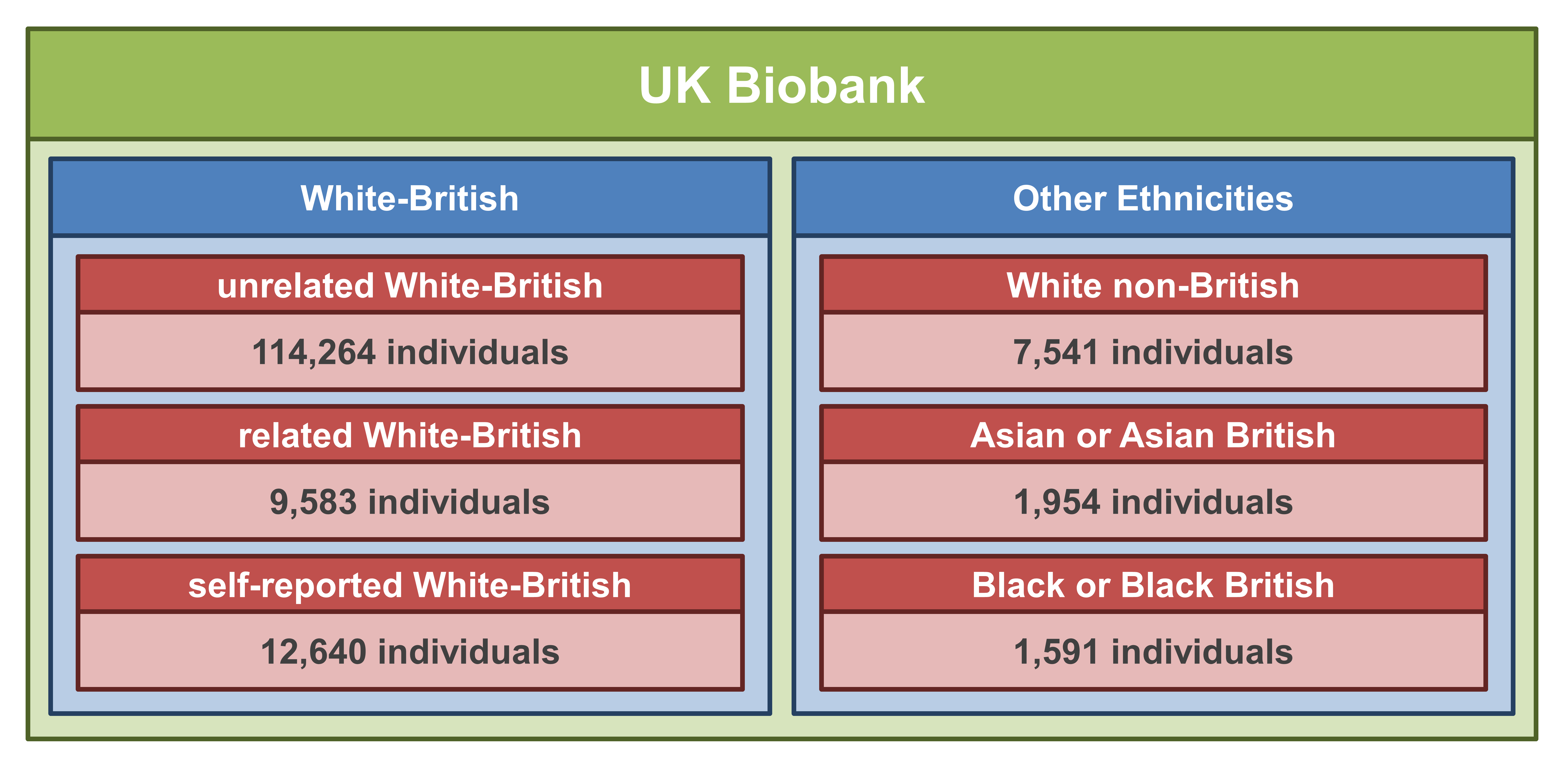

Supplement: S1 Fig — (PNG) [file pone.0166755.s001.png]

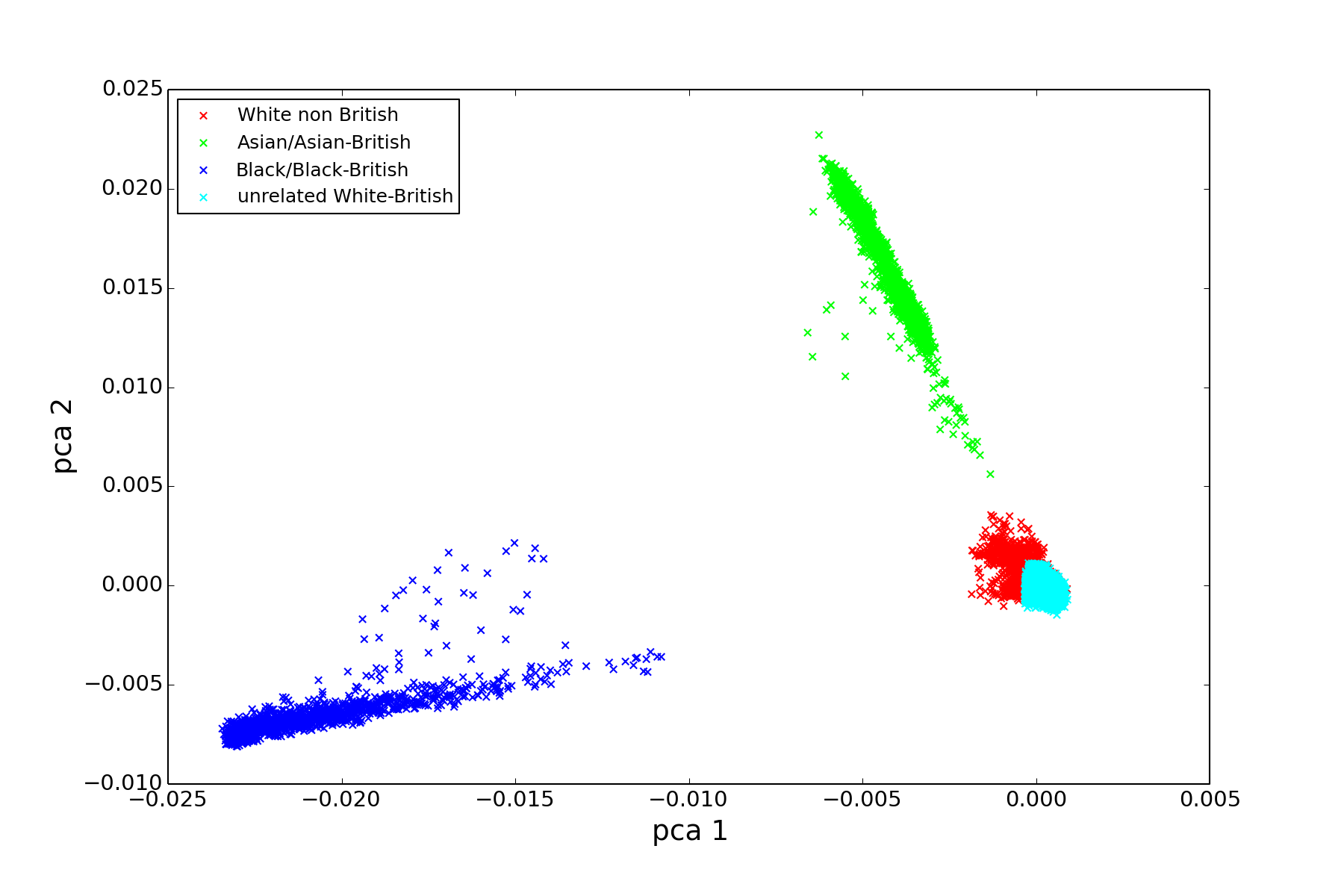

Supplement: S2 Fig — The individuals from different ethnic backgrounds are plotted using different colors. (PNG) [file pone.0166755.s002.png]

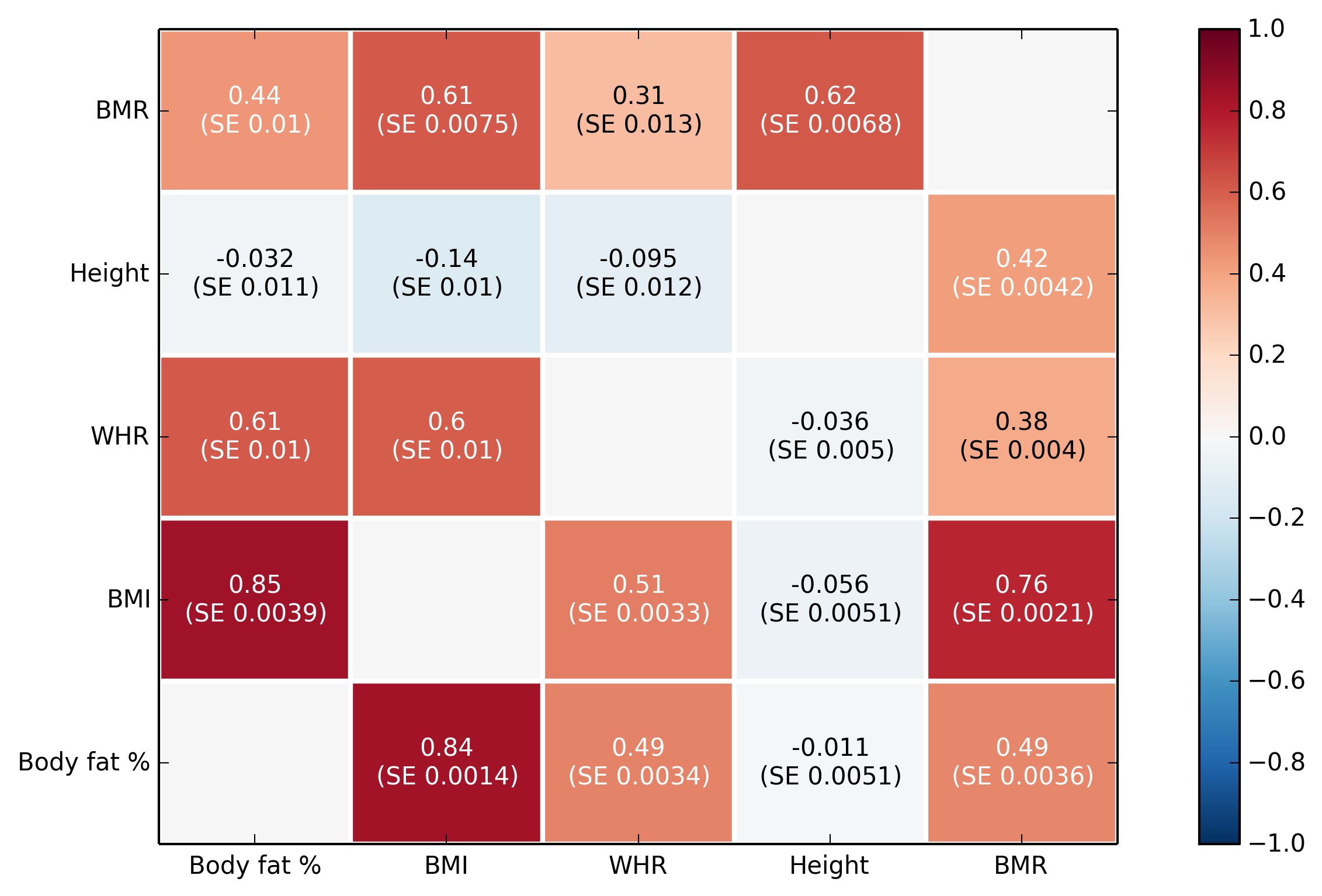

Supplement: S4 Fig — Genetic and environmental correlations displayed over and below the diagonal, respectively. (PNG) [file pone.0166755.s004.png]
