# Supplementary material for: Improved Genetic Profiling of Anthropometric Traits Using a Big Data Approach
Source: PLoS One. 2016 Dec 15;11(12):e0166755. doi: 10.1371/journal.pone.0166755 (PMC5157980; doi:10.1371/journal.pone.0166755)
Supplement: S1 Table — (DOCX) [file pone.0166755.s005.docx]

| **Traits** | **Prediction accuracy (SE)** |
| --- | --- |
| **Height** | 0.49 (0.01) |
| **Body fat percentage** | 0.26 (0.01) |
| **BMI** | 0.25 (0.01) |
| **WHR** | 0.20 (0.01) |
| **BMR** | 0.30 (0.01) |
