# Supplementary material for: Improved Genetic Profiling of Anthropometric Traits Using a Big Data Approach
Source: PLoS One. 2016 Dec 15;11(12):e0166755. doi: 10.1371/journal.pone.0166755 (PMC5157980; doi:10.1371/journal.pone.0166755)
Supplement: S2 Table — (DOCX) [file pone.0166755.s006.docx]

| **Traits** | **White non-British non-Irish** | **Irish** |
| --- | --- | --- |
| **Height** | 0.48 (0.46-0.51) | 0.49 (0.46-0.51) |
| **Body fat percentage** | 0.22 (0.2-0.25) | 0.26 (0.22-0.29) |
| **BMI** | 0.23 (0.2-0.26) | 0.26 (0.23-0.29) |
| **WHR** | 0.18 (0.15-0.2) | 0.2 (0.17-0.24) |
| **BMR** | 0.29 (0.26-0.32) | 0.33 (0.3-0.36) |
