# Supplementary material for: Improved Genetic Profiling of Anthropometric Traits Using a Big Data Approach
Source: PLoS One. 2016 Dec 15;11(12):e0166755. doi: 10.1371/journal.pone.0166755 (PMC5157980; doi:10.1371/journal.pone.0166755)
Supplement: S3 Table — (DOCX) [file pone.0166755.s007.docx]

| **Traits** | **Only common variants analysis (SE)** | **Common and rare variants analysis (SE)** | | |
| --- | --- | --- | --- | --- |
|  | **h2** | **h2 MAF0.001-0.01** | **h2 MAF0.01-0.05** | **h2 MAF0.05-0.5** |
| **Height** | 0.53 (0.0043) | 0.0067 (0.0013) | 0.072 (0.0041) | 0.5 (0.0045) |
| **Body fat**  **percentage** | 0.26 (0.0047) | 0.0024 (0.0015) | 0.022 (0.0044) | 0.25 (0.0048) |
| **BMI** | 0.26 (0.0046) | 0.0015 (0.0014) | 0.025 (0.0044) | 0.25 (0.0048) |
| **WHR** | 0.2 (0.0045) | 0.0013 (0.0014) | 0.022 (0.0044) | 0.19 (0.0047) |
| **BMR** | 0.31 (0.0047) | 0.0044 (0.0015) | 0.031 (0.0044) | 0.3 (0.0049) |
