# Supplementary material for: Improved Genetic Profiling of Anthropometric Traits Using a Big Data Approach
Source: PLoS One. 2016 Dec 15;11(12):e0166755. doi: 10.1371/journal.pone.0166755 (PMC5157980; doi:10.1371/journal.pone.0166755)
Supplement: S4 Table — (DOCX) [file pone.0166755.s008.docx]

| **Traits** | **MAF0.05-0.5** | **MAF0.01-0.05 + MAF0.05-0.5** | **MAF0.001-0.01 + MAF0.01-0.05 + MAF0.05-0.5** |
| --- | --- | --- | --- |
| **Body fat**  **percentage** | 0.27 (0.25-0.29) | 0.27 (0.25-0.29) | 0.27 (0.25-0.29) |
| **BMI** | 0.25 (0.24-0.27) | 0.26 (0.24-0.27) | 0.26 (0.24-0.27) |
| **WHR** | 0.2 (0.19-0.22) | 0.21 (0.19-0.22) | 0.21 (0.19-0.22) |
| **Height** | 0.51 (0.49-0.52) | 0.51 (0.5-0.52) | 0.51 (0.5-0.52) |
| **BMR** | 0.32 (0.31-0.34) | 0.32 (0.31-0.34) | 0.32 (0.31-0.34) |
