# Supplementary material for: Improved Genetic Profiling of Anthropometric Traits Using a Big Data Approach
Source: PLoS One. 2016 Dec 15;11(12):e0166755. doi: 10.1371/journal.pone.0166755 (PMC5157980; doi:10.1371/journal.pone.0166755)
Supplement: S5 Table — (DOCX) [file pone.0166755.s009.docx]

| **Traits** | **White non British** |
| --- | --- |
| **Height** | 0.5 (0.48-0.51) |
| **BMR** | 0.33 (0.31-0.35) |
