# Supplementary material for: Improved Genetic Profiling of Anthropometric Traits Using a Big Data Approach
Source: PLoS One. 2016 Dec 15;11(12):e0166755. doi: 10.1371/journal.pone.0166755 (PMC5157980; doi:10.1371/journal.pone.0166755)
Supplement: S6 Table — (DOCX) [file pone.0166755.s010.docx]

| **Traits** | **White non British** |
| --- | --- |
| **Body fat percentage** | 0.26 (0.24-0.29) |
| **BMI** | 0.26 (0.24-0.28) |
| **WHR** | 0.21 (0.19-0.23) |
